# Pediatric Teleradiology in Low-Income Settings and the Areas for Future Research in Teleradiology

**DOI:** 10.3389/fpubh.2014.00125

**Published:** 2014-08-21

**Authors:** Savvas Andronikou

**Affiliations:** ^1^Department of Radiology, Faculty of Health Sciences, University of the Witwatersrand, Johannesburg, South Africa

**Keywords:** teleradiology, tuberculosis, pulmonary, HIV infections, developing countries, resource allocation, cost-effectiveness, X-rays

## Abstract

Teleradiology is an established mechanism to overcome the lack of on-site radiologists and can benefit children in developing countries. In this “perspective” on teleradiology for pediatric care in underdeveloped countries, three low-cost teleradiology programs are discussed from experiences of one teleradiologist, in relation to previous publications on this subject. Key issues discussed include mechanisms for sustainability, cost-effectiveness, resources, and barriers to success. Reliance on each link of a telereading chain is highlighted as a constant source for concern.

## Introduction

In low-income settings, teleradiology has a significant role to play in the diagnosis and management of respiratory diseases in children. Teleradiology has been shown to improve the diagnosis of tuberculosis, especially in settings with a high burden of HIV infection (1).

Limited radiology services are a major obstacle to health care for sick children. Some sub-Saharan African countries have no radiologists in public service (1). This is when teleradiologic interpretation of diagnostic imaging can circumvent the need for on-site radiologists; children in underserved areas benefit from X-ray reports by expert radiologists, without having to travel and with quick results offering the opportunity for earlier and more appropriate treatment (1–3).

For a telereading program to be successful, it must be cost-effective for an underserved population, overcome technical, legal, and language barriers, and lead to improved outcomes sustainably. This paper describes my personal experience in three different teleradiology programs. I will highlight possibilities, imaging quality, and major obstacles to providing a sustainable service from the point of view of a pediatric radiologist, and make research recommendations that explore the expanding role of teleradiology for children in low-income settings.

## Three Different Experiences with Teleradiology

### Telereading for Médecins Sans Frontières

#### Intersectional radiologist and ad hoc MSF telereader

In 2009, I was employed by Médecins Sans Frontières (MSF) as “intersectional radiologist.” In addition to administrative tasks, I was mandated to promote the use of diagnostic imaging consultation through e-mail across all operational centers.

**Figure 1:**
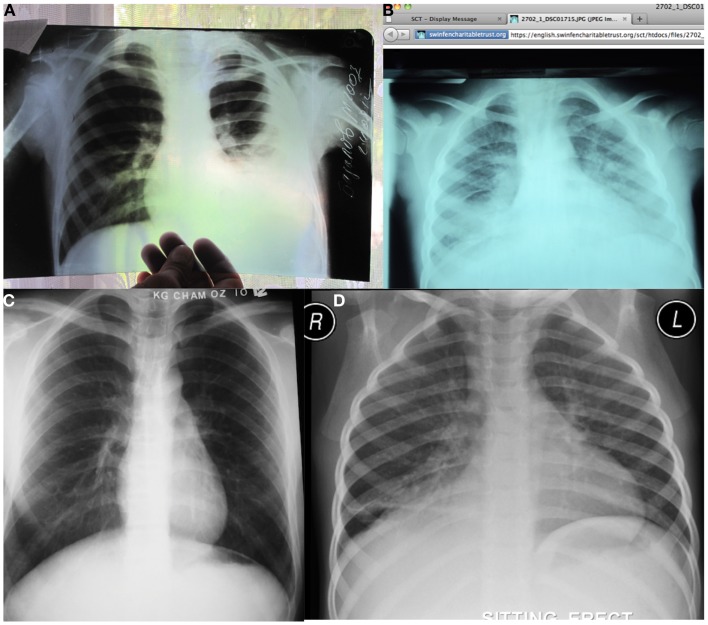
**A variety of pediatric telereading referrals**. **(A)** An X-ray referral from an MSF site in Tajikistan was received as a JPEG created by holding the hard copy X-ray up against a window and photographing it digitally. The curtains on the backdrop are seen through portions of the radiograph as a fine checker pattern. Although this was an inadequate X-ray referral the left-sided effusion is clearly visible. **(B)** Pediatric X-ray referral from the Collegium Telemedicum platform for MSF. The radiograph is of relatively poor quality as it is underinspired and the patient is markedly rotated to the left. In addition, the image has not been converted to gray scale. The right middle lobe air-space disease is visible but difficult to distinguish from density at the bases probably the result of poor inspiration. It is also difficult to comment on the presence of lymphadenopathy due to the vascular crowding, again due to under inspiration. **(C)** A radiograph referred from an MSF site in Cambodia is of higher quality, probably owing to the digital equipment available, which also avoids the quality losses from photographing or scanning hard copies. Note that positioning quality is improved with older patients but that on this occasion the right costophrenic angle has been cut off. **(D)** A digital X-ray referral from Khayelitsha District Hospital involves direct conversion of a DICOM image to JPEG for limiting file size and e-mailing through a control telereader to an expert pediatric radiologist. On this radiograph, a right lower lobe air-space disease process was reported but there were no features identified to suggest tuberculosis. The radiographic quality and labeling is excellent, as there are professionally trained radiographers working on-site.

I spent a significant portion of my time explaining that the lack of expertize in the interpretation of X-ray and ultrasound could be overcome through digitizing the images and referring them to an expert radiologist by e-mail. The process of digitizing hard copy film from existing X-ray units with wet developing was the first obstacle to overcome. Referrals gave me concerns both with regard to the expectations of the clinicians and the quality of the imaging (Figure 1A). A colleague receiving regular referrals from a pediatric MSF site using a digital unit in Liberia had more success and satisfaction, managing to assist the clinicians regularly with management changing diagnoses.

I re-oriented my efforts into educating control centers on the benefits of digital imaging; promoting CR conversions of existing units; establishing digitization guidelines for hard copy films; and improving imaging quality through site visits. Subsequent to my departure, two formal teleradiology pilot programs were set up.

#### Formal MSF telereading pilot projects

Médecins Sans Frontières teleradiology has evolved from an *ad hoc*, e-mail based X-ray consultation service, into a structured platform-based service. I have been pediatric teleradiologist for MSF using the Collegium Telemedicus platform (4), since its creation. Other telereaders also volunteer, at no cost to MSF, reporting X-rays from number of MSF field projects. Most MSF equipment is supplied by the local Ministry of Health, using hard copy film and wet developing. To enable telereading, hard copies were photographed against viewing boxes using a digital camera. JPEG images were transmitted via Collegium Telemedicus for reporting. More recent MSF X-ray equipment in Sub-Saharan Africa is digital and can be transmitted unchanged for telereading.

In 2012, 818 plain X-ray images from 14 sites were sent for teleradiology opinion. A significant proportion of these (36%) were pediatric cases. I have provided opinions on 72 pediatric imaging referrals from 24 July 2012 to April 2014 from Malawi, Central African Republic, Uganda, Guinea, Tajikistan, and Cambodia. These were mainly chest radiographs from HIV projects for diagnosing tuberculosis. The quality of radiographs varied from very poor (Figure 1B) to diagnostic (Figure 1C), yet I was able to assist the clinicians and engage in dialog with them, which was satisfying. I was also able to assist in the development of the diagnostic imaging manual for the organization.

### E-mail telereading controller and reader for WFPI/Khayelitsha project

The World Federation of Pediatric Imaging (WFPI) pilot telereading program was created to provide opinions on pediatric X-rays from the Khayelitsha District Hospital in the Western Cape of South Africa, in 2012. As outreach chairman for the society, I acted as manager of teleradiology for a 7-month period. The hospital itself is located in an informal township in the “Cape Flats” serving a population of 406,779 (2005), comprising fewer than 7% adults over 50 years old. Over 40% of the residents are younger than 19 years of age.

This is a new hospital where family physicians supervised by the district pediatrician care for children. Approximately one-third of the 1,000 annual imaging examinations at the hospital are pediatric X-rays. Prior to the pilot teleradiology project, radiographs were interpreted by the physicians. The X-ray equipment is new, and the on-site radiographers are well trained and produce high-quality radiographs.

As control teleradiologist, I distributed pediatric X-ray referrals to radiologists from a network of up to 50 volunteer pediatric radiologists. All volunteers spoke English and hailed from 17 different countries (USA, South Africa, Brazil, China, India, Colombia, Pakistan, Spain, the UK, Argentina, Bolivia, Cuba, Sri Lanka, Australia, Panama, New Zealand, and Italy).

**Figure 2:**
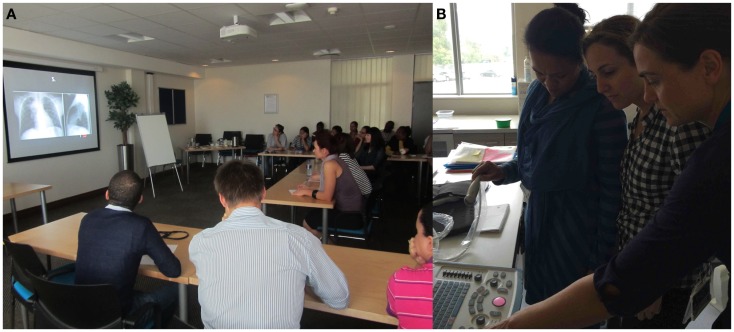
**Training of non-radiologists to interpret radiographs is part and parcel of telereading support and capacity development**. **(A)** A large group of family physicians participating in a pre- and post-training, pediatric X-ray reporting, self-assessment session, hosted by the visiting outreach radiologist from the South African Society of Pediatric Imaging acting (under the auspices of the World Federation of Pediatric Imaging). This program was run twice in parallel with the pilot telereading program, intended to increase capacity for X-ray interpretation on the ground. **(B)** Point of care mediastinal sonography for the diagnosis of tuberculosis being taught to a non-radiologist by a representative of Imaging the World in co-operation with the World Federation of Pediatric Imaging.

A total of 555 referrals and 1,106 radiographs were submitted for teleradiology opinion during the period 26 July 2012 to 3 March 2013. The majority of these were chest radiographs (75%), mainly for the evaluation of tuberculosis (Figure 1D). I reported 76 referrals, often having to fill in for a non-responding volunteer. In addition, I trained the clinicians on-site in the interpretation pediatric radiographs for the diagnosis of TB on two occasions (Figure 2A).

To ensure the sustainability of this program, an “institutional buddy” system is being tested in conjunction with Stanford University. Radiology opinions are provided for specific physician requests through e-mail. Stanford University through the Lucille Packard Children’s Hospital offers the service through its residents who provide opinions under the supervision of an experienced consultant. Only six radiographs have been reported so far in the first few months. Stanford University benefits by providing its residents with exposure to developing country pathology. This symbiotic relationship may prove to be the key to sustainability. The WFPI hopes to encourage other university hospitals to engage in such reciprocal systems.

### WFPI telereading of CT scans for the Indira Gandhi Children’s Hospital pilot

Lack of sub-specialist pediatric imaging expertize on-site, the availability of a CT scanner and the significant childhood population visiting the Indira Gandhi Children’s Hospital made this a feasible telereading pilot project for the WFPI. We faced several challenges in setting up teleradiology at this site. Lack of proximity of a WFPI member and the geographical distance between the contributors made the setup complex. Meetings are also difficult to schedule because of time zone differences, and communications are primarily through e-mail. We also needed more than e-mail as a platform for interpreting CT scans because of the large amount of images.

A telereading digital platform was created, but it took a long time to get the referring site and WFPI volunteers to install the software. The developers of the software have been helpful but they are distant to the site and are sometimes naïve to the restrictions and obstacles faced locally. The installation of the software requires administrative privileges and the information technology infrastructure of the department must be known for configuring proxy servers, etc. The volunteers’ information technology departments did not allow software installation in some instances, which meant that volunteers had to interpret the studies on their own personal computers. There are also cost implications with a current fee of 1.00€ (US $1.36) per uploaded study. Even though some cases have been referred successfully and reports have been provided, this telereading project is stuttering along. Lessons have been learnt regarding requirements for setting up new telereading software and with regard to having personnel on the ground to troubleshoot.

## Cost-Effectiveness

Most sustainable teleradiology programs have employed low-cost systems using “low-technology” methods (5). E-mail consultation is a simple solution that has proved to be effective, useful, and acceptable (2) with precedent in the successful Swinfen Charitable Trust projects operation over many years (3). E-mail is not recommended, however, as it is an unsecure, and the delivery mechanism cannot be guaranteed.

Zanaboni and Wootton identified 37 cross-border telemedicine services that used store and forward technology such as e-mail. This was the commonest form of telemedicine, using the Internet and a digital camera (3). An MSF project in Malawi used digitization of X-rays by photographing them using a digital camera and transmitting them electronically to a radiologist initially via e-mail (and subsequently through a web-based telemedicine service – Médecins Sans Frontières/Swinfen Charitable Trust). Reports were returned by the radiologist in the United States to the MSF physician free of charge by e-mail or entry on the telemedicine web site. Teleradiology changed patient management in some cases by reducing the time to a definite diagnosis and preventing misdiagnosis (1) without measurable cost. Another project in Ethiopia also used photographs of X-rays with a camera, “home-made” open source telemedicine software, and ran between 2004 and 2006 in 10 health care sites. This project was considered unsuccessful, as it was not sustainable. The authors noted problems common to Sub-Saharan African countries – low bandwidth, slow connections, and high service charges (2).

“Low-cost” digital X-ray devices are now available for low-resource settings, and these can significantly improve the quality of local X-ray images. Digital X-ray is a solution for low-resource countries because it eliminates film development and processing, and enables teleradiology (6). One project in Angola implemented digital radiology and provided teleradiology reports free of charge, by e-mail. The cost of purchasing the equipment in 2010 was 26,660 US$. It was calculated that the initial cost was paid back in 2 years by eliminating the need for expensive films and reagents (6).

## Barriers to the Practice of Teleradiology

The major limiting factors for many countries are low bandwidth, slow Internet connections, and high Internet service charges (2). To combat this, JPEG compression is used to decrease image file size but this in turn leads to poorer image quality. Nonetheless, several studies have demonstrated that JPEGs, such as used in a number of MSF projects, obtained by digital photography of radiographs are sufficient for diagnosis in most instances (1).

Language is cited as a potential limitation (5) as it can prevent teleradiologists from understanding key information and prevent the requesting physician from understanding the opinions offered. One solution to this is the use of an international panel of volunteers from all over the world as per the WFPI volunteer network.

Another limitation is the quality of the images referred for interpretation, because the radiologist is distant from the site where the imaging is performed and has little influence over the technologists performing the studies. For teleradiology to be effective, quality standards must be maintained (7). The image quality of hard copy radiographs is affected by factors such as poor equipment, substandard materials, and the inherent nature of the screen-film technology (7). The MSF quality assurance program found that those sites using film and chemistry for X-ray imaging demonstrated significantly more non-diagnostic images than those sites with digital imaging. Inadequate exposure was the most frequent problem (68%, *n* = 143). In all, 24% (*n* = 51) of film images demonstrated artifacts compared with 1% (*n* = 7) of digital images. The five MSF sites performing poorest for quality were all ministry of health facilities using film and chemistry (7).

## The Chain – Any Break in a Link Breaks the System

The quality of radiographs sent for a teleradiology opinion can be adversely affected by any part of a long chain of events. Programs converting hard copy into digital files via digital cameras create additional technical considerations. There is limited opportunity to improve the quality of a hard copy image if the primary radiograph is poor (6). Digital technology allows for a wider margin of radiographic error, makes post processing improvements possible, is simpler to use, obviating the need for development and processing, has less inherent artifact, and also simplifies the practice of teleradiology because images can be directly electronically converted to JPEG format (6). With a shorter chain of events, there are fewer possibilities for error, making this more desirable for limited resource settings.

An effective teleradiology operation also requires appropriately qualified radiologists to be available, and (usually) a mechanism for distributing incoming cases to the most appropriate specialist. This requires a vigilant teleradiology manager who has knowledge of the available human resources and understanding of the different skills of each radiologist.

The strength of the teleradiology chain is equivalent to its sustainability, i.e., the program “must be adopted into everyday practice and continue to function with high activity levels after any pilot funding runs out” (6). Success also depends on suitable governance, effective policy development, human resource management, and capacity building (2). Radiologists familiar with the local environment are a pre-requisite for project survival beyond the initial pilot phase (3).

## Future Research

Collecting and evaluating data from pilot projects are essential for future planning.

Important information includes the proportion of all pediatric examinations sent for teleradiology, the reasons for referral, the elapsed time from request to receipt of opinion, the number of requests not responded to, the effect on patient management, the volunteer teleradiologist workload, number of volunteers who left the project, and the image quality of the JPEGs. It is also extremely important to know the costs. Such research requires that projects have adequate mechanisms for capturing data.

An extension of telereading that requires more research is the interpretation of ultrasound imaging performed by non-radiologists. Point of care sonography, especially for diagnosis of TB and pneumonia in children, is a novel way of overcoming the expense, complexity, and radiation risk associated with X-ray imaging (8). This is in addition to other indications for assessing the kidneys, abdomen, pleural effusions, and intracranial compartment in neonates. Ultrasound is ideally suited for pediatric imaging at the point of care. However, because it is operator-dependent, operators often pay little attention to routine anatomical image acquisitions and labeling and there has been little consideration for telereading. With a little effort to standardize imaging and train on-site personnel in orientation and technique, opinion of ultrasound images through telereading is possible. Standardized imaging protocols already exist for trauma (FAST), abdominal HIV, and TB (FASH) and even cranial ultrasound, but the thrust has been for the person at the point of care to make a focused assessment for immediate management or triage (8).

Imaging The World (ITW) has paved the way for telereading ultrasound imaging performed by personnel who have no anatomical knowledge but understand surface landmarks. These “sonographers” produce standardized sweeps based on surface landmarks that are compressed and sent over the Internet for telereading. WFPI has partnered with ITW to produce pediatric protocols. In addition, a research project testing the diagnostic capability of the telereaders’ sweep of the mediastinum against an expert free hand sonographer is in progress in Cape Town South Africa for the diagnosis of TB in children (Figure 2B). This research based at the Red Cross Children’s Hospital (University of Cape Town) is being expanded for the diagnosis of pneumonia. The research possibilities of telereading for point of care sonography are extensive and are relatively safe due to the nature of sonography. However, image compression techniques and free telereading platforms need to be researched further to meet the limitations of bandwidth and Internet speeds in developing countries.

## Conclusion

Although teleradiology is a viable option to alleviate radiologist shortages in underserved areas, there are many challenges to designing an adequate teleradiology chain. Teleradiology is possible through simple e-mail of JPEG images or through more sophisticated means and can assist doctors working in developing countries with expert support to manage sick children. Pediatric radiology volunteers have a responsibility to examine further avenues of telereading for point of care sonography through research and training programs.

## Conflict of Interest Statement

The author declares that the research was conducted in the absence of any commercial or financial relationships that could be construed as a potential conflict of interest.
